# Effects of scenario‐based attribution on collective emotions and stigma toward persons with COVID‐19: A cross‐sectional survey

**DOI:** 10.1002/hsr2.1039

**Published:** 2023-01-09

**Authors:** Hye In Boo, Yun‐Kyeung Choi

**Affiliations:** ^1^ Department of Psychology, BK21 Education & Research Team for Disaster and Trauma Intervention Keimyung University Daegu Korea

**Keywords:** attribution, collective emotions, COVID‐19, lung cancer, social stigma

## Abstract

**Background and Aims:**

During this COVID‐19 pandemic, many people experience and share emotions such as fear, anxiety, sadness, anger, and disgust, which can be regarded as collective emotions. This study investigated the effects of scenario‐based attribution for serious diseases on collective emotions and social stigma.

**Methods:**

Participants were 297 healthy adults who met two conditions: (1) not having tested positive for COVID‐19 (including their family members or close friends) and no experience of self‐quarantine; and (2) not having been diagnosed with lung cancer, and not having family members or close friends diagnosed with it. Three hundred participants were recruited, through a company conducting online surveys. A total of 297 data sets were analyzed, excluding data supplied by three participants who might have responded unreliably to the filler question. Scenarios were recorded according to attribution type (internal vs. external) and disease (COVID‐19 vs. lung cancer). A 2 × 2 factorial design was used, whereby participants were randomly assigned to one of four conditions.

**Results:**

The COVID‐19 condition showed higher scores on the perceived risk and fear of the disease compared to the lung cancer one. The COVID‐19/internal attribution condition showed the highest scores for fear and anger toward scenario characters, and the lung cancer/external attribution condition showed higher sympathy scores than other conditions. Although attribution to COVID‐19 was not directly related to social stigma, it could evoke negative emotions toward infected people.

**Conclusion:**

The findings suggest that attributions of serious diseases such as COVID‐19 to infected persons can influence collective emotions and the level of social stigma associated with the disease. Attention to the collective emotions and stigma associated with disease is a key component for communities and countries to recover from and respond to its impacts.

## 1 INTRODUCTION

The global pandemic triggered by the spread of the coronavirus disease 2019 (COVID‐19) has led to “collective emotions” such as fear, anxiety, sadness, anger, and disgust.^1^ Collective emotions have been defined as emotions that are shared by a large number of individuals in a certain society^2^ and/or as emotions felt by individuals as a result of their membership in a group or society.^3^ Negative collective emotions not only induce psychological distress in the infected but also lead to stigma, social criticism, and discrimination. These negative emotional responses and stigma can be influenced by attribution style, which is a method of reasoning that seeks to find the cause of one's own and other's behaviors and their consequences.^4^ Attribution to the same event may depend on the actor's or observer's point of view,^5^ which is called the actor‐observer bias.^6^ The actor‐observer bias is described as the tendency to judge other's negative behavior (e.g., nonadherence to quarantine guidelines) more harshly than one's own negative behavior.^7^ Because it is highly likely to be attributable to internal attributes such as the actor's personality, moral responsibility, attitude, and characteristics (i.e., internal attribution) and people have little prior knowledge of an actor's life experiences or typical behavior and situational forces in the observer's point of view.^7^ In a study by Mantler et al.^8^ participants read a brief description of a male with a serious disease (i.e., HIV/AIDS vs. lung cancer) and rated their controllability, responsibility, and blame for the disease. Regardless of disease type, controllability, responsibility, and blame were higher in the condition of internal attribution than in external attribution, which could be associated with the actor‐observer attribution bias. These results showed that internal attribution to the disease can lead to blame being placed on and stigma associated with the person with the disease.

According to the risk assessment hypothesis, perceived risk can influence fear and lead to discriminatory behavior toward the person with the disease, irrespective of the type of attribution to the cause of the disease.^9^ Previous studies^8^, ^10^ showed that the stigma associated with HIV/AIDS was significantly higher than for tuberculosis (TB) and severe acute respiratory syndrome (SARS) and the correlation between attributions (i.e., controllability, responsibility, and blame) and stigma was significant. The attribution model, including the sequential paths from controllability through responsibility and blame, to public stigma, was supported across HIV/AIDS, SARS, and TB.^10^ However, the public perceived that people with HIV/AIDS were more responsible and blameworthy than those with other infectious diseases. TB and SARS are more infectious but treatable compared to HIV/AIDS, whereas the latter is more lethal and has a negative image related to moral responsibility, so the fear of HIV/AIDS infection and the likelihood of infection through simple contact can be overestimated.^10^ Uncertainty over information about the disease and repeated exposure to the media leads to an overestimation of the risk of infectious diseases and causes anxiety, panic, and social stigma.^11^ It is hypothesized that this will be applied to the early stages of COVID‐19, wherein uncertainty is very high.

The purpose of this study was to investigate the effects of attribution to COVID‐19 infection on collective emotions and social stigma, based on the determinants propounded by Mantler et al.^8^ and Mak et al.^10^ Specifically, four types of scenarios were recorded according to the disease (COVID‐19 vs. lung cancer) and attribution (internal vs. external).^8^ Lung cancer is a respiratory disease similar to COVID‐19, and an individual's lifestyle and behavioral habits such as smoking are closely related to the onset of lung cancer, indicating a higher perceived stigma than for other types of cancer.^12^, ^13^ Therefore, it was set as a comparative condition. In this study, as a collective emotion, emotional response was defined as “sympathy” toward the person with the disease, in addition to negative emotions such as fear and anger toward the person with the disease. In addition, as the COVID‐19 pandemic continued, the fear of the disease itself was also measured.

## 2 METHODS

### 2.1 Participants

Participants were adults aged 18 years or older who met two conditions: (1) not having tested positive for COVID‐19 (including their family members or close friends) and no experience of self‐quarantine; and (2) not having been diagnosed with lung cancer, and not having family members or close friends diagnosed with it. Three hundred participants were recruited nationwide in proportion to the distribution of the Korean population census, through an online survey company (http://www.invight.co.kr), and randomly assigned to one of four scenarios, that is, COVID‐19/internal attribution (35 males, 40 females), COVID‐19/external attribution (39 males, 35 females), lung cancer/internal attribution (34 males and 39 females), and lung cancer/external attribution (40 males and 35 females). A filler question to check the reliability of responses (e.g., “How many times did you wear a mask in the past month?”) was included. A total of 297 data sets were analyzed, excluding data supplied by three participants who might have responded unreliably to the filler question. The demographic characteristics of the participants are shown in Table 1. Participants ranged from 18 to 65 years (M = 42.8, SD = 9.53), half were female (50.2%, male = 49.8%) and most were married/living together 66.6%. Others were unmarried 32.3%, divorced 3.7% and bereaved 2.0%. The number of household members was three or more 68.0%, two‐people 18.2%, and one‐person 13.8%. Living together with the elderly was 25.6%. Participants' highest level of education was a college/university undergraduate degree (72.7%), postgraduate degree (13.8%), and high school and below (13.5%). Most were employed (83.5%) and 10.8% were housewives. Most of the participants were nonreligious (59.9%), followed by Christianity (19.2%), Buddhism (10.4%), Catholicism (10.1%), and others (0.3%). Smoking experience was none 52.5%, quitting smoking 24.6%, and currently smoking 22.9%.

**Table 1:** Demographic and test for homogeneity according to disease type and conditions of attribution in the scenario

	Covid‐19 scenario						Lung cancer scenario						
	Internal attribution (*n* = 75)			External attribution (*n* = 74)			Internal attribution (*n* = 73)			External attribution (*n* = 75)			
Variable	*n*	*M*	*SD*	*n*	*M*	SD	*n*	*M*	SD	*n*	*M*	SD	Statistic test
Sex (Male: female)	35:40			39:35			34:39			40:45			χ^2^ (3, *N* = 297) = 1.222
Age		43.12	9.70		43.24	9.85		42.42	9.06		42.42	9.06	F (3, *N* = 297) = 0.165
Marital status (unmarried/divorced/bereaved: married/living together)	29:46			26:48			30:43			28:47			*χ* ^2^ (3, *N* = 297) = 0.583
The number of household members (one: two: three or more)	8:14:53			11:11:52			15:14:44			7:15:53			*χ* ^2^ (3, *N* = 297) = 5.573
Older family members (living together: apart)	19:56			18:56			21:52			18:57			χ^2^ (3, *N* = 297) = 0.551
Years of education (12 or less: 13~16: 17 or more)	10:51:14			10:57:7			15:52:6			5:56:14			χ^2^ (3, *N* = 297) = 11.004
Employment (employed: unemployed/housewife)	14:61			12:62			12:61			11:64			*χ* ^2^ (3, *N* = 297) = 0.443
Religion (none: having a religion)	48:27			37:37			49:24			45:30			χ^2^ (3, *N* = 297) = 5.129
Smoking (none: quit smoking: smoking)	42:18:15			38:17:19			39:14:20			37:24:4			χ^2^ (3, *N* = 297) = 4.740
Anxiety		3.56	3.74		4.77	4.53		4.32	4.76		3.33	4.17	F (3, *N* = 297) = 1.783
Subjective health status		2.84	.83		2.83	.95		2.87	.78		2.73	.81	F (3, *N* = 297) = 0.394
The locus of control													
Internality		37.45	5.39		36.64	5.92		38.00	5.77		36.94	5.49	*F* (3, *N* = 297) = 0.231
Chance		29.65	5.36		29.00	6.51		32.09	5.68		29.63	5.26	*F* (3, *N* = 297) = 1.083
The powerful others		34.84	6.24		33.26	6.37		35.07	6.96		32.54	6.78	F (3, *N* = 297) = 0.470
Knowledge about the disease		39.60	17.97		39.32	14.27		39.72	17.40		35.73	18.39	*F* (3, *N* = 297) = 2.297

### 2.2 Measures

#### 2.2.1 Sociodemographic variables

The sociodemographic characteristics of the participants consisted of nine items. The first set of items were sex, age, marital status, years of education, employment, and religion; the items were the number of household members, whether older family members lived together, and smoking,^14^ which were identified as characteristics affecting fear of infection and emotional distress.^15^, ^16^, ^17^, ^18^, ^19^

#### 2.2.2 Controlled variables

##### Anxiety

The Generalized Anxiety Disorder 7‐item scale (GAD‐7)^20^, ^21^ is a self‐report scale designed to assess the frequency at which a participant has been disturbed by each symptom over the past 2 weeks. The GAD‐7 was measured on a 4‐point Likert scale for each item ranging from 0 (not at all) to 3 (nearly every day), with an optimal cut‐off value of 10 points for the GAD. This study used the Korean version of GAD‐7,^22^ which added the phrase “in relation to COVID‐19” to control the COVID‐19 related “anxiety” of participants. The Korean version of the GAD‐7^22^ showed excellent internal consistency (α = 0.93), and the internal consistency of this study was 0.90.

##### Subjective health status

Subjective health status is an indicator of an individual's overall condition or quality of life, including physical, mental, and social aspects. Subjective health status was evaluated with one item: “How do you feel about your health in general compared to others of your age?” Participants were asked to respond on a 5‐point Likert scale from 1 (very healthy) to 5 (very unhealthy). The higher the score, the worse the perceived health status. In this study, subjective health status was used as one of the control variables according to a previous study.^23^

##### The locus of control

The Internal Powerful Others and Chance (IPC) Scale is a self‐report scale for assessing locus of control,^24^, ^25^ which consists of three subscales, the Internality (I)—the extent to which people believe they have personal control over their lives, the Powerful Others (P)—the belief that other people control events in one's life, and the Chance (C)—belief that luck or fate affects experiences. This scale has a total of 24 items with 8 items for each subscale. Participants responded using a 7‐point Likert scale with response choices ranging from 1 (strongly disagree) to 7 (strongly agree). This study used the Korean version of the IPC^1^ to precontrol the attribution styles of participants. In the study of Choi and Oh^26^ reported the internal consistency of each subscale as follows: 0.65 (I), 0.74 (P), and 0.68 (C) in the Korean version of IPC. The internal consistency of this study was 0.71 (I), 0.70 (P), and 0.78 (C) for each subscale, respectively.

##### Knowledge about the disease

This multiple‐choice questions were developed by the authors of this study based on the “knowledge about the disease” scale used by the study of Mak et al.^10^ This 10‐item questionnaire asked about knowledge of COVID‐19 (five items) and lung cancer (five items), and was constructed based on the most common misconceptions about the two diseases and information provided by the Korea Disease Control and Prevention Agency. The items were classified into categories of infection/incidence paths, symptoms, and treatment of disease. A higher proportion of correct responses indicated better knowledge of the disease.

#### 2.2.3 Dependent variables

##### Attribution of the disease

The “attribution of the disease” scale^10^ was translated into Korean and modified to include COVID‐19 and lung cancer scenarios by the authors of this study. Participants were asked how much they agreed on the cause of the disease in terms of controllability, responsibility, and blame in the scenario. The participants were asked to respond on a 6‐point Likert scale ranging from 1 (strongly disagree) to 6 (strongly agree). Higher scores indicated greater internal controllability, personal responsibility, and more blame for the disease associated with people infected with COVID‐19 or lung cancer.

##### Perceived risk of the disease

The Perceived Risk Scale^27^ was modified and used for COVID‐19 and lung cancer in this study. Participants were asked to respond, on a 7‐point Likert scale ranging from 0 (not at all) to 6 (strongly agree), to two questions about the severity of the impact and potential risk of the disease to themselves and their families. The internal consistency of the study was 0.88.

##### Fear of the disease

With reference to SARS‐related studies,^19^ this study used five questions measuring the fear of COVID‐19 or lung cancer (e.g., “I am afraid I will get COVID‐19 or lung cancer” or “I am afraid my family will get COVID‐19 or lung cancer”). Participants were asked to answer on a 4‐point Likert scale ranging from 0 (not at all) to 3 (strongly agree). The internal consistency of the present study was 0.84.

##### Emotional responses toward the scenario character

Emotional responses toward the scenario character were measured using a total of 10 items composed of adjectives expressing fear, anger, and sympathy. The “fear” subscale was measured by threatened, terrified, and scared, and the anger subscale was measured by aggravated, angered, and irritated, which were part of the Attribution Questionnaire‐27 (AQ‐27).^28^ A “sympathy” subscale was added to reflect the preliminary survey on feeling sympathy for the scenario character, which included adjectives such as sympathetic, sorry, pitiable, and compassionate. Participants were asked to respond to all questions on a 4‐point Likert scale ranging from 0 (not at all) to 3 (strongly agree). In this study, emotional responses were measured before and after the scenario presentation. The pre‐emotional response was measured by the emotions currently experienced by the participants, and the post‐emotional response was measured as the emotions toward the scenario character. The pre‐and postinternal consistency of each subscale of fear, anger, and sympathy were 0.93, 0.91; 0.89, 0.93; and 0.61, 0.78, respectively. As for the score of emotional responses toward the scenario character, the difference obtained by subtracting the pre‐ from the postscore was used for statistical analysis.

##### Stigma toward the disease

The “Stigma toward the Disease Scale” (SDS)^10^ was used to measure stigma toward people with the disease. The SDS consists of a total of 14 items, and each item is answered on a 6‐point Likert scale ranging from 1 (strongly disagree) to 6 (strongly agree). A higher score indicated a higher level of stigma toward people with the disease. The original SDS consisted of questions about the stigma toward those who recovered from SARS, but in this study, the questions were modified and used for people who recovered from COVID‐19, or those with lung cancer. The Korean version of the SDS (K‐SDS) was translated into Korean by the authors of this study. An exploratory factor analysis with principal axis factoring and direct oblimin (oblique) rotation was performed on 14 items of the K‐SDS. As a result, three factors were derived, and each factor was named stigmatization (6 items), rejection (5 items), and distancing (3 items). The internal consistencies of this study were 0.93, 0.77, and 0.83 for each subscale, respectively.

#### 2.2.4 Four types of scenarios

Based on the study by Mantler,^8^ four scenarios were specified according to disease type (COVID‐19 vs. lung cancer) and attribution type (internal vs. external). Each of the four scenarios had an equal number of words in Korean. The scenarios used in this study are as follows.

Example of the COVID‐19 scenario:
The following is the case of Minjae (33‐year‐old) who tested positive for COVID‐19. He is an office worker at Company “A.” He was spending most of his time at home, adhering to social distancing. As the trend of the number of confirmed cases subsided a little despite the ongoing spread of COVID‐19 in the community, *he was relieved and began to make appointments with his friends. He took the subway to meet his close friends after a long time. He felt stifled wearing a mask, so he wore his mask around his chin in the subway and did not continue to wear a mask in the dining pub* (internal attribution) […the company “A” recommended that employees go to work again. The distance between the desks in the office was too small, and he had to face many colleagues and clients while wearing a mask during work. It is not known if ventilation and disinfection are ensured regularly in his office (external attribution)].^1^ Recently, he started to experience mild pain in his throat and a mild fever. He visited the local screening center and underwent a COVID‐19 test. He tested positive for the coronavirus.
Example of lung cancer scenario:
The following is the case of Minjae (33‐year‐old) who was diagnosed with early‐stage lung cancer. He is an office worker at Company “A.” He spends most of his day at work, going to work in the morning, and leaving the office late in the evening, like a normal office‐goer. *Minjae, who has been smoking since high school, enjoys smoking in the smoking room with his co‐workers after lunch at a restaurant. In addition, if he loses his concentration while working, he goes to the company smoking room with his co‐workers to smoke, and smokes at home after work. He smokes more than one pack per day* (internal attribution). [*Minjae, who does not smoke, has constantly been submitting, for 6 years after joining, a suggestion to the company to prevent the spread of tobacco smoke in the company's smoking rooms. Minjae made the suggestion to company since he had a hard time working due to cigarette smoke, but there was no respite* (external attribution)].^1^ Recently, after undergoing a physical screening test, he reported abnormal findings in his lungs, and he underwent a thorough medical examination at a local university hospital. The medical examination revealed that Minjae was diagnosed with lung cancer (early stages).

### 2.3 Ethics, permissions, and procedure

**Figure 1:**
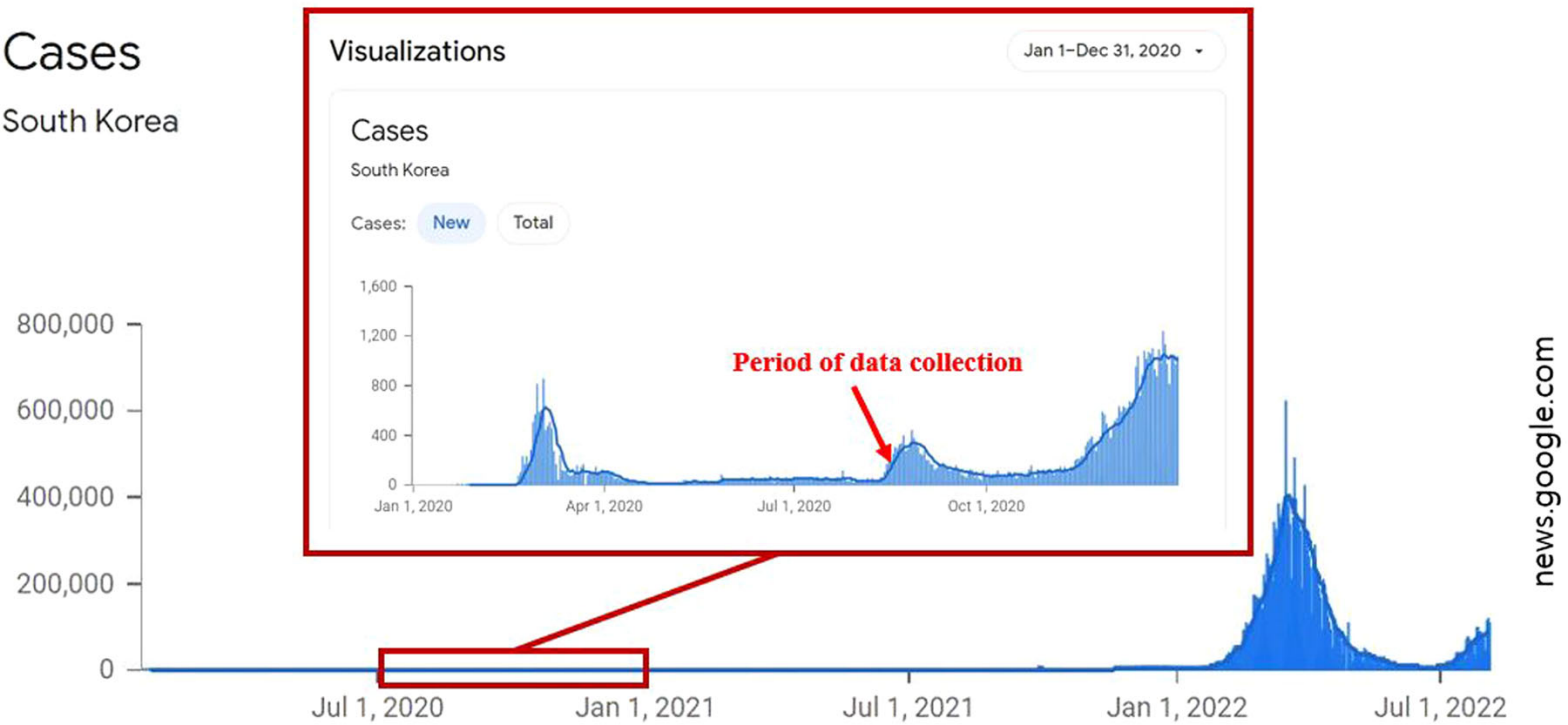
Period of data collection

This study was conducted with the approval of the Institutional Review Board of Keimyung University (IRB No. 40525‐202007‐HR‐038‐04). Participants indicated consent form before completing the survey of this study. News at that time reported cases where one person was infected within the office and leading to a group infection and the public felt threatened by such news reports. The scenarios were reflected publics' attitudes toward COVID‐19 infected individuals at that time. The lung cancer scenario was constructed as similar as possible to the COVID‐19 scenario. Four scenarios were written according to disease type and attribution type by the first author, and reviewed by 16 graduate students majoring in clinical psychology and the 2nd author. Sixteen graduate students were asked to rate the degree of whether the scenario was perceived as internal or external attribution on an 11‐point Likert scale ranging from −5 (internal attribution) to +5 (external attribution). As a result, the mean and *SD* of each scenario condition are as follows; COVID‐19/internal attribution (*M* = −3.38, *SD* = 0.44), COVID‐19/external attribution (*M* = 3.75, *SD* = 0.27), lung cancer/internal attribution (*M* = −4.38, *SD* = 0.20), and lung cancer/external attribution (*M* = 4.69, *SD* = 0.15). Although the internal or external attribution was rated to be higher in the lung cancer condition compared to the COVID‐19 condition, the direction of attribution in each scenario was evaluated as appropriate. Data were collected from an online survey company. The period of survey was November 10–13, 2020 (Figure 1), when the number of confirmed COVID‐19 cases in Korea increased again after the second wave of infections. The daily counts of confirmed cases during the period of study were 100, 146, 143, and 191, respectively. First, participants were randomly assigned to one of the four scenario conditions. Then, they read the scenario from an observer's point of view, and responded to a series of questionnaires. It took approximately 20–30 min to complete the questionnaires.

### 2.4 Study design and data analysis

This study used a 2 (disease: COVID‐19 vs. lung cancer) ×2 (attribution: internal vs. external) factorial design. The dependent variables were perceived risk, fear of the disease, emotional responses toward the scenario character (i.e., *Δ*fear, *Δ*anger, and *Δ*sympathy), and stigma toward the diseases. To examine the prior homogeneity of each condition, a *χ* ^2^ test or ANOVA was performed on sociodemographic information and controlled variables including anxiety, self‐rated health, and locus of control (i.e., internality, powerful others, and chance). In addition, a two‐way ANOVA was performed to test the difference in dependent variables according to disease and attribution type.

## 3 RESULTS

### 3.1 Test for homogeneity between conditions

Table 1 shows that there were no significant the differences between four conditions for all sociodemographic variables, anxiety as measured by the GAD‐7, subjective health status, knowledge about the disease, and internality, chance and powerful others as measured by the locus of control (all *p*s > 0.05).

### 3.2 Comparisons of internal attribution

Table 2 presents comparisons of internal attribution (controllability, responsibility, and blame) scores between four conditions. The responsibility score in the COVID‐19 condition was significantly higher than that in the lung cancer condition (*p* < 0.05) but the difference in controllability and blame scores between the two conditions was not significant. The internal attribution condition showed significantly higher scores of controllability, responsibility and blame than external attribution (all *p*s < 0.001). Only the interaction effect of disease×attribution for controllability score was significant [*F*(1, 293) = 9.25, *p* = 0.003, partial *ɳ* ^2^ = 0.031]. Analysis of simple main effects showed that controllability score in COVID‐19 scenario was higher than that of lung cancer scenario in the condition of internal attribution [*F*(1, 293) = 12.25, *p* = 0.001, partial *ɳ* ^2^ = 0.040], but that there was no difference of controllability score between two disease scenarios in the condition of external attribution [*F*(1, 293) = 0.63, *p* = 0.426, partial *ɳ* ^2^ = 0.002].

**Table 2:** Means, standard, and two‐way ANOVA according to disease type and conditions of attribution in the scenario

	Covid‐19 scenario				Lung cancer scenario						
	Internal attribution (*n* = 75)		External attribution (*n* = 74)		Internal attribution (*n* = 73)		External attribution (*n* = 75)				
Variable	*M*	*SD*	*M*	*SD*	*M*	*SD*	*M*	*SD*	*F_D_*	*F*_*A*_	*F*_*D* ×_ *_A_*
Controllability	5.01	1.18	2.96	1.42	4.25	1.25	3.13	1.46	3.68	104.88***	9.25**
Responsibility	5.16	0.89	2.64	1.26	4.95	0.80	2.35	1.17	4.30*	446.09***	0.09
Blame	4.68	1.11	2.28	1.14	4.73	0.90	2.11	1.12	0.28	405.91***	0.80
Perceived risk of disease	7.20	0.37	6.82	0.37	6.73	0.37	5.77	0.37	4.25*	3.23	0.61
Fear of disease	9.05	0.42	8.77	0.42	6.62	0.41	6.87	0.40	28.92**	0.00	0.44
Emotional responses toward the scenario character^a^											
*Δ*Fear	2.68	0.34	0.93	0.34	0.30	0.34	0.71	0.34	15.00**	3.98*	10.25**
*Δ*Anger	3.47	0.33	−0.62	0.33	0.82	0.33	0.40	0.33	6.15*	47.50***	31.39***
*Δ*Sympathy	1.15	0.33	2.77	0.33	2.27	0.34	4.19	0.33	14.67***	28.36***	0.19
Stigma toward the disease	37.71	1.29	36.78	1.29	40.48	1.30	37.13	1.29	1.46	2.73	0.88
Stigmatization	12.81	0.69	12.47	0.70	14.62	0.70	12.17	0.69	1.17	4.02*	2.29
Rejection	14.73	0.55	14.31	0.55	17.22	0.55	17.00	0.55	22.25***	0.34	0.03
Distancing	10.16	0.40	10.00	0.40	8.64	0.41	7.96	0.40	19.61***	1.10	0.43

^a^ Emotional responses toward the scenario character is calculated as the pre‐ from the postscore.

* *p* < 0.05;

** *p* < 0.01;

*** *p* < 0.001.

### 3.3 Comparisons of perceived risk and fear of disease

In the scores for perceived risk and fear of disease, the interaction of disease × attribution and the main effect of attribution type were not significant. Participants in the COVID‐19 condition showed higher scores for perceived risk (*p* < 0.05) and fear of disease (*p* < 0.01) than those in the lung cancer condition.

### 3.4 Comparisons of emotional responses toward the scenario character

There were significant interaction effects of the disease×attribution type on *Δ*fear and *Δ*anger toward the scenario character [*F*(1, 293) = 10.25, *p =* 0.002, partial *ɳ* ^2^ = 0.034; *F*(1, 293) = 31.39, *p =* 0.000, partial *ɳ* ^2^ = 0.097]. As a result of simple main effect analysis, *Δ*fear in COVID‐19 scenario were higher than those of lung cancer scenario in the condition of internal attribution [*F*(1, 293) = 24.93, *p =* 0.000, partial *ɳ* ^2^ = 0.078], but not in the condition of external attribution. The *Δ*anger toward the scenario character in COVID‐19 scenario was higher than that of lung cancer scenario in both internal [*F*(1, 293) = 32.56, *p =* 0.000, partial *ɳ* ^2^ = 0.100] and external [*F*(1, 293) = 4.89, *p* = 0.028, partial *ɳ* ^2^ = 0.016] attribution conditions. Participants in the external attribution and lung cancer conditions showed higher sympathy scores toward the scenario character compared to those in the internal attribution and COVID‐19 conditions, respectively (all *p*s < 0.001).

### 3.5 Comparisons of stigma toward COVID‐19 and lung cancer

The main effects of the disease [*F*(1, 293) = 1.46, *p* = 0.228, partial *ɳ* ^2^ = 0.005] and the attribution [*F*(1, 293 )= 2.73, *p* = 0.099, partial *ɳ* ^2^ = 0.009], and the interaction effect of the disease × attribution types on social stigma were not significant [*F*(1, 293) = 0.88, *p* = 0.349, partial *ɳ* ^2^ = 0.003]. Additional analyses of the subscales of social stigma showed that stigmatization scores were higher in the internal attribution condition than in the external attribution condition (*p* < 0.05), and rejection and distancing scores were higher in the COVID‐19 condition than in the lung cancer condition (*p*s < 0.001).

## 4 DISCUSSION

The purpose of this study was to examine the effects of COVID‐19 on the collective emotions and stigma experienced by a community and its members. Participants read a scenario of a character with COVID‐19 or lung cancer, and rated the attribution, perceived risk, fear of disease, emotional responses toward the scenario character, and stigma toward the disease from an observer's perspective.

First, when the character in the scenario became ill because of their actions and not due to external causes, the participants perceived the character to have control and therefore be responsible or blameworthy for the disease. These results are consistent with the actor‐observer attribution bias.^5^ Participants considered those with COVID‐19 to be more responsible for their disease than those with lung cancer. In the internal attribution condition, COVID‐19 was also regarded as much more controllable than lung cancer. These results seem to relate to the current influence of COVID‐19, although lung cancer is far more lethal.^29^, ^30^ Nonpharmaceutical interventions such as social distancing and taking a mask, have been an important means through which to control the spread of COVID‐19 viruses.^31^ High adherence to guidelines was associated with a high perception of risk of contracting COVID‐19,^31^, ^32^ seeing others' adhere,^31^, ^33^ and collective culture.^34^ Collective culture such as Korea may show high adherence to such guidelines, and an individual or groups perceived as being responsible for the origin and spread of infection are often severely blamed.^31^, ^35^

Second, participants responded to the COVID‐19 condition by citing a greater perceived risk of disease and greater fear of disease compared to the lung cancer condition. This result is consistent with previous studies^36^, ^37^ that show an infectious disease has an uncertain route of transmission and is perceived as more uncontrollable, so risks can be overestimated and fear can be greatly induced. Although lung cancer^29^, ^30^ is a more serious and fatal disease that is difficult to detect in early stages and the survival rate is lower compared to cases of other types of cancer, the general public perceives the risk of COVID‐19 more seriously.

The results of excessive risk perception and fear of COVID‐19 can be explained by the availability heuristic, that is, the phenomenon in which more easily imaginable events are perceived as more likely to occur,^38^ and collective emotions.^39^ The news media broadcasted the number of daily confirmed cases and deaths from COVID‐19 and disclosed the route of the infections in confirmed cases. The public in the community was exposed to text messages (e.g., the number of daily confirmed cases in their local area) from local government agencies several times a day and would have perceived excessive the risk of being infected themselves by the availability heuristic.^38^ As such, excessive exposure to information related to COVID‐19 induced cognitive biases, which might have influenced the risk appraisal process^40^ and higher levels of anxiety.^41^, ^42^ Moreover, the risk of infectious diseases might have been overestimated and the fear and anxiety of the public increased in a situation where the number of confirmed cases in Korea decreased and subsequently increased again and no treatment or vaccine has been developed.^11^ Fear and anxiety during the COVID‐19 pandemic^12^ can form an emotional climate and collective emotions through social sharing, which motivates people to take self‐protective actions and measures.^43^

Fear and anger toward the scenario character increased further, and sympathy decreased in the condition that showed COVID‐19 infection to be attributable to irresponsible behavior, which is partially consistent with the attribution theory.^44^ Fear and anger toward the scenario character in internal attribution conditions increased further in the COVID‐19 scenario, but sympathy toward the scenario character was higher in the lung cancer than in the COVID‐19 condition. Moral responsibility is imposed on those diagnosed with COVID‐19 and not following infectious disease prevention guidelines, thus increasing the possibility of anger toward the infected person^8^ and fear associated with the need for self‐protection.^43^ If the disease is caused by an external factor, sympathy toward a person with the disease may increase in terms of the greater negative loss from lung cancer than from COVID‐19.^44^ Absolution from moral responsibility when the disease is due to an uncontrollable factor (e.g., biological cause) may also explain the increased sympathy for the person with the disease.^45^

Finally, the stigma toward the scenario character was not significantly different by disease and attribution types. This result suggests that there was no difference in stigma according to the type of disease (COVID‐19 vs. lung cancer) or the perceived cause of the disease (internal vs. external attributions), but it does not mean that there was no stigma towards individuals with the disease. Stigma in the context of health is particularly prominent during highly contagious epidemics and pandemics^10^, ^46^ and the felt stigma of people infected can obstruct treatment‐seeking. Above all, the stigmatization of people infected and groups or regions affected (e.g., Wuhan coronavirus^47^) goes beyond discrimination and can lead to crime (e.g., racism towards Asians).^48^ It was reported that a 77 percent increase from 2019 to 2020 in hate crimes against Asians in the US.^49^ Although about 30% of nonsmokers can develop lung cancer,^50^ the patients are stigmatized smokers. Lung cancer survivors, with or without smoking, blame themselves for the disease by internalizing stigma, which can lead to social isolation and psychological distress.^51^

Additional results showed characteristic patterns of stigma according to disease and attribution. The score of stigmatization subscale was higher for the internal attribution than the external attribution condition. These results are consistent with previous studies^10^, ^52^ which showed that internal attribution towards individuals with a disease is closely related to social stigma. When stigma was compared in lung cancer and COVID‐19 conditions, the rejection score was higher for the lung cancer condition and the distancing score was higher for the COVID‐19 condition. These results may be influenced by the perception that individuals, their loved ones, or anyone close to them could easily be infected with COVID‐19 through only one contact, unlike the diagnosis of lung cancer which worsens over a period of time.

This study suggests that efforts are needed to reduce cognitive biases (e.g., actor‐observer bias, stigma) in the face of the threat of an infectious disease. For example, it is required careful use of terminology, the transmission of science‐based facts about a disease and its prevention and treatment, and a positive social environment of empathy and care.^53^ This study has several limitations. First, as a large number of questions in an online survey could increase the fatigue of the respondents, the attribution of the disease scale used in this study measures controllability, responsibility, and blame with a single item. Also, the questions of the SDS^8^ were different according to disease conditions—“a person diagnosed with lung cancer” and “a person who recovered from COVID‐19.” Had the stigma associated with a person infected with COVID‐19 been measured, it is possible that the results would have been different. Internal consistencies of the Korean version of IPC were low,^24^ although coefficients of all subscales in our study were higher than 0.70. In future research, it will be necessary to use measurement tools with good reliability. Second, the data for this study were collected at a time when the number of COVID‐19 cases repeatedly rose and fell, so the public was still experiencing anxiety and fear of infection. Although respondents in which the participants, their family members, and close acquaintances had COVID‐19 or lung cancer were excluded through the screening process, the ongoing COVID‐19 pandemic may have affected the survey responses. So, fear and anger toward individuals with COVID‐19 were greater than those with lung cancer, especially when disease was attributed to their misbehavior, even though lung cancer is a more fatal disease than COVID‐19, at least in Korea.^27^, ^28^ It is necessary to conduct a replication study in the future after the pandemic comes to an end. Third, this study was cross‐sectional study showing differences among attributions, collective emotions and stigma according to scenario‐based attribution and disease conditions. This study is thought to reflect collective emotions and stigma in the initial stage of the COVID‐19 pandemic. Future studies are needed to investigate long‐term change process of collective emotions and stigma according to the mutation of the COVID‐19 virus and to test effect of intervention strategies to prevent stigma.

## 5 CONCLUSION

Despite these limitations, this study focused on collective emotions shared by the community and its members during the COVID‐19 pandemic and measured attitudes and emotions toward the character using scenarios to increase ecological validity. More negative emotional reactions are being presented to those infected with COVID‐19 due to the individual's failure to comply with quarantine rules, which can influence collective emotions and social stigma during pandemics. Collective emotions can affect the thinking and behavior of members of society, and may lead to hatred, prejudice, stigma, and discrimination toward individuals or groups infected with the COVID‐19 virus. Considering that there is an attribution bias from the observer's point of view, the importance of accurate information processing and communication should be emphasized.

## AUTHOR CONTRIBUTIONS

**Hye In Boo**: Conceptualization; data curation; formal analysis; funding acquisition; investigation; methodology; writing – original draft; writing – review & editing. **Yun‐Kyeung Choi**: Funding acquisition; project administration; supervision; writing – review & editing.

## CONFLICT OF INTEREST

The authors declare no conflict of interest.

## TRANSPARENCY STATEMENT

The lead author Yun‐Kyeung Choi affirms that this manuscript is an honest, accurate, and transparent account of the study being reported; that no important aspects of the study have been omitted; and that any discrepancies from the study as planned (and, if relevant, registered) have been explained.

## Data Availability

The data sets analyzed in the paper are available from the author or corresponding author upon readers' requests.

## ENDNOTES

1 The parts in italics vary depending on whether the conditions is an internal or external attribution type.

## References

[1] Hermans D , Rimé B , Mesquita B . Changing Emotions. Psychology Press; 2013.

[2] Stephan WG , Stephan CW . An Integrated Threat Theory of Prejudice: reducing Prejudice and Discrimination. Lawrence Erlbaum Associates; 2000.

[3] Smith ER . Social Identity and Social Emotions: toward New Conceptualizations of Prejudice. Academic Press; 1993.

[4] Heider F . The Psychology of Interpersonal Relations. Psychology Press; 2013.

[5] Jones EE , Nisbett RE . The actor and the observer: divergent perceptions of the causes of behavior. Preparation of this paper grew out of a workshop on attribution theory held at University of California. Lawrence Erlbaum Associates, Inc; 1987:79‐94.

[6] Wilson SR , Levine KJ , Cruz MG , Rao N . Attribution complexity and actor‐observer bias. J Soc Behav Pers. 1997;12(3):709‐726.

[7] Kulibert D , Thompson AE . Stepping into their shoes: reducing the actor‐observer discrepancy in judgments of infidelity through the experimental manipulation of perspective‐taking. J Soc Psychol. 2019;159(6):692‐708. 30614776 10.1080/00224545.2018.1556575

[8] Mantler J , Schellenberg EG , Page JS . Attributions for serious illness: are controllability, responsibility and blame different constructs? Can J Behav. 2003;35(2):142‐152.

[9] Corrigan P , Markowitz FE , Watson A , Rowan D , Kubiak MA . An attribution model of public discrimination towards persons with mental illness. J Health Soc Behav. 2003;44(2):162‐179. 12866388

[10] Mak WWS , Mo PKH , Cheung RYM , Woo J , Cheung FM , Lee D . Comparative stigma of HIV/AIDS, SARS, and tuberculosis in Hong Kong. Soc Sci Med. 2006;63(7):1912‐1922. 16766106 10.1016/j.socscimed.2006.04.016 PMC7115765

[11] Cheng C . To be paranoid is the standard? Panic responses to SARS outbreak in the Hong Kong special administrative region. Asian Perspect. 2004;28(1):67‐98.

[12] ElseQuest NM , LoConte NK , Schiller JH , Hyde JS . Perceived stigma, self‐blame, and adjustment among lung, breast and prostate cancer patients. Psychol Health. 2009;24(8):949‐964. 20205038 10.1080/08870440802074664

[13] LoConte NK , ElseQuest NM , Eickhoff J , Hyde J , Schiller JH . Assessment of guilt and shame in patients with non‐small‐cell lung cancer compared with patients with breast and prostate cancer. Clin Lung Cancer. 2008;9(3):171‐178. 18621628 10.3816/CLC.2008.n.026

[14] Kim BH . Osteoporosis knowledge and health behavior in Korean adult men. Unpublished master's thesis, Ewaha Womans University, Seoul. 2010.

[15] Chua SE , Cheung V , McAlonan GM , et al. Stress and psychological impact on SARS patients during the outbreak. Can J Psychiatry. 2004;49(6):385‐390. 15283533 10.1177/070674370404900607

[16] Hawryluck L , Gold WL , Robinson S , Pogorski S , Galea S , Styra R . SARS control and psychological effects of quarantine, Toronto, Canada. Emerging Infect Dis. 2004;10(7):1206‐1212. 10.3201/eid1007.030703 PMC3323345 15324539

[17] Lau ALD , Chi I , Cummins RA , Lee TMC , Chou KL , Chung LWM . The SARS (severe acute respiratory syndrome) pandemic in Hong Kong: effects on the subjective wellbeing of elderly and younger people. Aging Ment Health. 2008;12(6):746‐760. 19023726 10.1080/13607860802380607

[18] Maunder R , Lancee W , Balderson K , et al. Long‐term psychological and occupational effects of providing hospital healthcare during SARS outbreak. Emerging Infect Dis. 2006;12(12):1924‐1932. 10.3201/eid1212.060584 PMC3291360 17326946

[19] Nickell LA , Crighton EJ , Tracy CS , et al. Psychosocial effects of SARS on hospital staff: survey of a large tertiary care institution. Can Med Assoc J. 2004;170(5):793‐798. 14993174 10.1503/cmaj.1031077 PMC343853

[20] García‐Campayo J , Zamorano E , Ruiz MA , et al. Cultural adaptation into Spanish of the generalized anxiety disorder‐7 (GAD‐7) scale as a screening tool. Health Qual Life Outcomes. 2010;8(1):1‐11. 20089179 10.1186/1477-7525-8-8 PMC2831043

[21] Spitzer RL , Kroenke K , Williams JBW , Löwe B . A brief measure for assessing generalized anxiety disorder: the GAD‐7. Arch Intern Med. 2006;166(10):1092‐1097. 16717171 10.1001/archinte.166.10.1092

[22] Seo JG , Park SP . Validation of the generalized anxiety disorder‐7 (GAD‐7) and GAD‐2 in patients with migraine. J Headache Pain. 2015;16(1):1‐7. 26596588 10.1186/s10194-015-0583-8 PMC4656257

[23] Lee DH , Kim JY , Kang HS . The emotional distress and fear of contagion related to Middle East respiratory syndrome (MERS) on general public in Korea. Korean J Psychol Gen. 2016;35(2):355‐383.

[24] Levenson H . Activism and powerful others: distinctions within the concept of internal‐external control. J Pers Assess. 1974;38(4):377‐383.

[25] Levenson H . Differentiating among internality, powerful others, and chance. In: Lefcourt HM , ed. Research With the Locus of Control Construct. Academic Press; 1981:15‐63. http://learningstorm.org/wp-content/uploads/2020/04/ipcscales.pdf

[26] 최자연 JY , Kyung‐Ja oh Oh . The effects of socially prescribed perfectionism on anger‐in expression: the mediating effects of external locus of control. Korean J Health Psychol. 2015;20(1):161‐173.

[27] Zhao X , Leiserowitz AA , Maibach EW , RoserRenouf C . Attention to science/environment news positively predicts and attention to political news negatively predicts global warming risk perceptions and policy support. J. Commun. 2011;61(4):713‐731.

[28] Crespo M , PérezSantos E , Muñoz M , Guillén AI . Descriptive study of stigma associated with severe and persistent mental illness among the general population of Madrid (Spain). Community Ment Health J. 2008;44(6):393‐403. 18437569 10.1007/s10597-008-9142-y

[29] Mani D , Haigentz M , Aboulafia DM . Lung cancer in HIV infection. Clin Lung Cancer. 2012;13(1):6‐13. 21802373 10.1016/j.cllc.2011.05.005 PMC3256276

[30] Park JY , Jang SH . Epidemiology of lung cancer in Korea: recent trends. Tuberc Respir Dis. 2016;79(2):58‐69. 10.4046/trd.2016.79.2.58 PMC4823185 27064578

[31] Williams SN , Armitage CJ , Tampe T , Dienes KA . Public perceptions of non‐adherence to pandemic protection measures by self and others: a study of COVID‐19 in the United Kingdom. PLoS One. 2021;16(10):e0258781. 34710125 10.1371/journal.pone.0258781 PMC8553167

[32] Carlucci L , D'Ambrosio I , Balsamo M . Demographic and attitudinal factors of adherence to quarantine guidelines during COVID‐19: the Italian model. Front Psychol. 2020;11:e559288. 10.3389/fpsyg.2020.559288 PMC7609562 33192820

[33] Coroiu A , Moran C , Campbell T , Geller AC . Barriers and facilitators of adherence to social distancing recommendations during COVID‐19 among a large international sample of adults. PLoS One. 2020;15:e0239795. 33027281 10.1371/journal.pone.0239795 PMC7540845

[34] Bavel JJV , Baicker K , Boggio PS , et al. Using social and behavioural science to support COVID‐19 pandemic response. Nat Hum Behav. 2020;4(5):460‐471. 32355299 10.1038/s41562-020-0884-z

[35] Smith LE , Potts HWW , Amlȏt R , Fear NT , Michie S , Rubin GJ . Holding a stigmatizing attitude at the start of the COVID‐19 outbreak: a cross‐sectional survey. Br J Health Psychol. 2022;27(2):588‐604. 34606149 10.1111/bjhp.12564 PMC8646234

[36] Herek GM , Capitanio JP . Public reactions to AIDS in the United States: a second decade of stigma. Am J Public Health. 1993;83(4):574‐577. 8460738 10.2105/ajph.83.4.574 PMC1694493

[37] Herek GM , Capitanio JP , Widaman KF . Stigma, social risk, and health policy: public attitudes toward HIV surveillance policies and the social construction of illness. Health Psychol. 2003;22(5):533‐540. 14570537 10.1037/0278-6133.22.5.533

[38] Tversky A , Kahneman D . Availability: a heuristic for judging frequency and probability. Cogn Psych. 1973;5(2):207‐232.

[39] Bar‐Tal D , Halperin E , De Rivera J . Collective emotions in conflict situations: societal implications. Journal of Social Issues. 2007;63(2):441‐460.

[40] Ropeik D . The consequences of fear: our modern world is a risky place and evokes many well‐founded fears. But these fears themselves create a new risk for our health and well‐being that needs to be addressed. EMBO Rep. 2004;5(S1):S56‐S60. 15459737 10.1038/sj.embor.7400228 PMC1299209

[41] Turner MM , Rimal RN , Morrison D , Kim H . The role of anxiety in seeking and retaining risk information: testing the risk perception attitude framework in two studies. Hum Commun Res. 2006;32(2):130‐156.

[42] Jagtap S , Shamblaw AL , Rumas R , Best MW . Information seeking and health anxiety during the COVID‐19 pandemic: the mediating role of catastrophic cognitions. Clin Psychol Psychother. 2021;28(6):1379‐1390. 34734452 10.1002/cpp.2684 PMC8652628

[43] Valenzano A , Scarinci A , Monda V , et al. The social brain and emotional contagion: COVID‐19 effects. Medicina. 2020;56(12):640. 33255569 10.3390/medicina56120640 PMC7760735

[44] Weiner B . Judgments of Responsibility: a Foundation for a Theory of Social Conduct. Guilford Press; 1995.

[45] Kim JH , Kim JH . Effective message strategy for charitable donation campaign with mortality salience. Korean J Advert. 2015;26(5):27‐57.

[46] Des Jarlais DC , Galea S , Tracy M , Tross S , Vlahov D . Stigmatization of newly emerging infectious diseases: AIDS and SARS. Am J Public Health. 2006;96(3):561‐567. 16449597 10.2105/AJPH.2004.054742 PMC1470501

[47] Di Y , Li A , Li H , et al. Stigma toward Wuhan people during the COVID‐19 epidemic: an exploratory study based on social media. BMC Public Health. 2021;21(1):1958. 34715825 10.1186/s12889-021-12001-2 PMC8554505

[48] Cheah CSL , Wang C , Ren H , Zong X , Cho HS , Xue X . COVID‐19 racism and mental health in Chinese American families. Pediatrics. 2020;146(5):e2020021816. 32873719 10.1542/peds.2020-021816

[49] Findling M , Blendon RJ , Benson J , Koh H . COVID‐19 has driven racism and violence against Asian Americans: perspectives from 12 national polls. Health Aff Forefr. 2022;4(12):05‐16. 10.1377/forefront.20220411.655787

[50] Park YS . Clinical Characteristics and Risk Factor of Lung Cancer in Korean Never Smoker. The Graduate School Seoul National University; 2022.

[51] Webb LA , McDonnell KK , Adams SA , Davis RE , Felder TM . Exploring stigma among lung cancer survivors: a scoping literature review. Oncol Nurs Forum. 2019;46:402‐418. 31225843 10.1188/19.ONF.402-418

[52] Cogan JC , Herek GM . Stigma. In: Smith RA , ed. The Encyclopedia of AIDS: A Social, Political, Cultural, and Scientific Record of the HIV Epidemic. Fitzroy Dearborn; 1998:466‐467.

[53] Zhou M . COVID‐19‐related stigma and its impact on psychological distress: a cross‐sectional study in Wuhan, China. Health Sci Rep. 2022;5(5):e758. 35949673 10.1002/hsr2.758 PMC9358535

